# 3D-Printable Oxygen- and Drug-Carrying Nanocomposite Hydrogels for Enhanced Cell Viability

**DOI:** 10.3390/nano12081304

**Published:** 2022-04-11

**Authors:** Ravi Kumar, Nermin Seda Kehr

**Affiliations:** 1 Physikalisches Institute, Westfälische Wilhelms-Universität Münster, Wilhelm-Klemm-Straße 10, 48149 Münster, Germany; ravikapoorsaini@gmail.com; 2 Center for Soft Nanoscience (SON), Westfälische Wilhelms-Universität Münster, Busso-Peus-Straße 10, 48149 Münster, Germany

**Keywords:** NC hydrogel, drug-delivery, oxygen-carrying hydrogels, 3D-printing

## Abstract

Nanocomposite (NC) hydrogels have been widely studied due to their tunable biochemical/ physical properties for tissue engineering and biomedical applications. Nanoparticles (NPs) that can carry bioactive hydrophilic/hydrophobic molecules and provide sustained release within hydrogels are an ideal all-in-one-platform for local drug delivery applications. Dual delivery of different bioactive molecules is desired to achieve synergetic therapeutic effect in biomedical applications. For example, the co-administration of drug molecules and oxygen (O_2_) is an ideal choice to improve cell viability, while reducing the harmful effects of hypoxia. Therefore, we prepared drug-loaded O_2_-carrying periodic mesoporous organosilica (PMO-PFC) NPs and their 3D-printable hydrogel precursors based on gelatin methacryloyl (GelMa) to fabricate 3D-scaffolds to improve cell-viability under both normoxia (21% O_2_) and hypoxia (1% O_2_) conditions. We used rutin as the hydrophobic drug molecule to demonstrate that our O_2_-carrying PMO-PFC NPs can improve hydrophobic drug loading and their sustained delivery over 7 days, while supporting sustained O_2_-delivery for 14 days under hypoxia conditions. Furthermore, the fibroblast cells were interacted with NC hydrogel scaffolds to test their impact on cell-viability under both normoxia and hypoxia conditions. The improved rheological properties suggest the prepared NC hydrogels can be further tested or used as an injectable hydrogel. The improved mechanical properties and 3D printability of NC hydrogels indicate their potential use as artificial tissue constructs.

## 1. Introduction

Nanocomposite (NC) hydrogels are crosslinked three-dimensional (3D) polymer networks embedded with nanoparticles (NPs) and have been used in tissue engineering and biomedical applications [1,2]. NC hydrogels mimic the extracellular matrix (ECM) environment, and tunable biochemical/physical properties can control cell functions such as cell adhesion, migration, proliferation, and differentiation. NC hydrogels are stimuli-responsive, hydrophilic, biocompatible, biodegradable and have excellent physical properties, such as mechanical toughness, elasticity, resistance against compression, and a high degree of swelling-deswelling. Several types of NPs (for example, carbon-based NPs, inorganic NPs, metal/metal oxide NPs, etc.) have been combined by physical or chemical interactions with polymeric chains of hydrogels, which results in novel properties in electronics, biosensors and biomedical applications [1,2,3,4,5,6]. By tailoring functionality of embedded NPs, NC hydrogels have opened new opportunities to develop advance biomaterials for tissue engineering and controlled drug delivery. The embedded NPs can serve as effective drug-carriers for both hydrophilic and hydrophobic drug molecules. The loading efficiency and controlled release of drugs can also be enhanced by the porous structure of hydrogels and large surface area and pores of NPs.

Numerous state-of-the-art approaches were demonstrated to fabricate NC hydrogels for controlled and site-specific drug delivery [7,8,9]. For example, Hou et al. [10] prepared a NC hydrogel using pH-responsive graphene oxide (GO) encapsulated with an anti-cancer drug (curcumin), thereby showing site-specific release to the proximal colon. In another example, Pacelli et al. [11] prepared a GelMa-based hydrogel embedded with nanodiamonds as a drug-carrier (dexamethasone, Dex), to promote the osteogenic differentiation of human adipose stem cells (hASCs). Zhao et al. [12] described Fe_3_O_4_ NPs incorporated into magnetic field-responsive NC hydrogels, thereby demonstrating the controlled release of bovine serum albumin (BSA) as the model drug. Alvarez et al. [13] described NC hydrogels consisting of silica NPs and collagen loaded with gentamicin/rifamycin drugs for wound-healing dressings and also investigated antibacterial activity. Motealleh et al. described alginate-based hydrogels made of periodic mesoporous organosilica with an antibacterial tetracycline (Tet) drug. The 3D-printed hydrogels showed a release of Tet for seven days and enhanced fibroblast cell proliferation [14]. Several studies also demonstrated the advanced application of NC hydrogels as local drug delivery systems due to their injectability and in situ forming abilities [8,15,16,17]. Furthermore, NC hydrogels can be used to fabricate 3D-scaffolds with tunable porosity and structure that are similar to tissue-like constructs due to their shear thinning and viscoelastic properties [18,19,20]. NPs that can carry bioactive molecules and provide sustained biomolecule release within hydrogels are ideal all-in-one-platforms for local drug delivery applications. Therefore, NC hydrogels are promising 3D functional artificial tissue constructs for drug delivery, and able to improve cellular attachment, proliferation, differentiation, and migration.

Rutin (quercetin-3-O-rutinoside) is a naturally occurring bioflavonoid found in vegetables and fruit. It has low solubility in water, thus showing poor stability and bio-availability [21]. Rutin, however, has various pharmacological activities (for example, antibacterial, antiprotozoal, antitumor, anti-inflammatory, antiallergic, antiviral etc.) [22]. Rutin-coated nanosystems have been demonstrated in various therapeutic objectives and have shown enhanced aqueous solubility, bioavailability and stability [23,24,25,26]; however, there are few studies conducted into loading rutin into hydrogels [27,28,29].

O_2_ is the most vital nutrient for cell survival and signal cascading to regulate cellular activities. The hypoxia condition occurs when there is insufficient (lower than normal oxygen level) and heterogeneous oxygen distribution within the body tissues, which leads to cell apoptosis, tissue necrosis, transplantation failures, and failures in tissue formation. Over the years, researchers demonstrated different approaches to O_2_-releasing hydrogels (either by introducing O_2_-releasing molecules within hydrogels or by embedding O_2_-releasing nanoparticles within NC hydrogels) to provide sufficient O_2_ to the tissues [30,31,32]. Commonly used O_2_-releasing materials are solid inorganic peroxides (for example, calcium peroxide and sodium percarbonate), hydrogen peroxide, and perfluorocarbons (PFCs). Alemdar et al. [32] prepared a GelMa-based hydrogel incorporated within calcium peroxide for O_2_ delivery in cardiac cells and demonstrated reduced hypoxia-induced cell death by limiting the necrosis. Kang et al. [33] investigated oxygen-generating alginate (OGA) hydrogel as a bioactive acellular matrix for enhanced wound healing. Patil et al. [34] demonstrated the use of methacrylamide chitosan modified with perfluorocarbon chains (MACF) to construct hydrogel dressings for treating dermal wounds. In another example, Li et al. [35] constructed hydrogels consisting of perfluorocarbons conjugated to methacrylamide chitosan to promote stem cell proliferation. Park et al. [36] reported a hyperbaric oxygen-generating (HOG) hydrogel consisting of thiolated gelatin and calcium peroxide, which promoted wound healing and neovascularization. However, these engineered functional tissues have low oxygen diffusion for in vitro and in vivo applications. In a scaffold, O_2_ can diffuse a limited distance of 100–200 μm so providing sufficient O_2_ to bigger non-vascularized scaffolds is an issue for in vivo application. The non-homogenous distribution of O_2_ through the non-vascularized scaffolds can also limit the repair to damaged tissue. Therefore, the incorporation of O_2_-releasing materials within scaffolds is suggested to provide sufficient O_2_ to the cells to maintain metabolic activity during the period of blood vessel ingrowth and repair of tissue. Co-delivery of different bioactive molecules is desired to achieve synergetic therapeutic effects in biomedical applications [37,38,39]. For example, the co-administration of drug molecules and oxygen (O_2_) is an ideal choice to improve cell viability, while reducing the harmful effects of hypoxia. The co-administration of O_2_ and drug molecules via NPs into NC hydrogels and the subsequent impact on cell viability have been studied only by a few researchers. For example, Newland et al. [40] described the simultaneous release of oxygen and doxorubicin from a gellan gum hydrogel loaded with calcium peroxide and the chemotherapeutic drug doxorubicin under normoxia and hypoxia. Motealleh et al. [41] prepared an alginate-based periodic mesoporous organosilica-consisting NC hydrogel to improve O_2_ release under the hypoxia condition, enhance healthy cell viability and decrease the viability of malignant and immortal cells in the presence of an anti-cancer drug.

We, therefore, aim to generate drug molecules loaded with O_2_-carrying NPs and their 3D-printable hydrogel precursors to fabricate 3D-scaffolds to improve cell viability under both normoxia and hypoxia conditions. To achieve this, we will use rutin as the hydrophobic drug molecule to demonstrate that our O_2_-carrying organic-inorganic NPs can improve hydrophobic drug loading and its sustained delivery over seven days, while supporting sustained O_2_-delivery for 14 days under hypoxia conditions. Recently, we demonstrated the preparation of rutin-loaded O_2_-carrying NPs, and their impact on fibroblast and Colo 818 cell viability under normoxia and hypoxia conditions on a 2D-cell culture plate [42]. A 3D biomaterial network can mimic the 3D tissue environment better than a 2D-cell culture plate [43]. Therefore, in the current study, we use similar NPs to generate injectable NC hydrogels to fabricate 3D-scaffolds to improve cell viability in 3D biomaterial network. We synthesized the oxygen-carrying nanoparticles (PMO-PFCs) and the surface of the PMO-PFCs was functionalized with a hydrophobic drug (rutin). Rutin-coated PMO-PFCs were further coated with a biodegradable and cell-adhesive bipolymer poly-d-lysine (PDL) for stability. Later, rutin-coated PMO-PFCs were incorporated into a gelatin methacryloyl (GelMa) -based hydrogel network. The NC-hydrogel scaffolds were prepared by lyophilization and measured for their capability to release oxygen under normoxia and hypoxia conditions. The drug release (rutin) profile was also observed at different pH for seven days. To demonstrate the enhanced cell viability under normoxia and hypoxia conditions, the NC-hydrogel scaffolds were interacted with the fibroblast cells. The morphology of the cells was examined by nuclei- and actin-staining after the cell experiment. The NC hydrogels were also characterized for rheological and mechanical properties. To summarize, drug-coated NC hydrogel shows sustained O_2_ over a period of 14 days and drug release at different pH values for seven days. The antioxidant effect of rutin, O_2_ release, and cell-adhesive coating (PDL) on PMO-PFCs slightly supported the cell viability under the hypoxia condition.

## 2. Materials and Methods

### 2.1. Materials

Hexadecyltrimethylammonium bromide (CTAB, 98%), [1H,1H,2H,2H perfluorooctyltriethoxysilane (PFC)], [1,2-bis(triethoxysilyl)ethane (BTEE, 96%)], Hoechst 33342 nuclei dye, [2′,7′-dichlorodihydrofluorescein diacetate (DCFHDA)], trypsin, poly-d-lysine (PDL), alginic acid sodium salt, powder gelatin (from porcine skin), methacrylic anhydride (MA), N-vinylcoprolactane (VC), eosin Y, triethanolamine (TEA), and the WST-1 assay were ordered from Sigma-Aldrich, Darmstadt, Germany. Rutin-trihydrate was purchased from Roth GmbH, Karlsruhe, Germany. Ammonia (32%), ethanol (absolute, for analysis) and hydrochloric acid (32%, for analysis) were bought from Merck, Darmstadt, Germany. Phalloidin Alexa Fluor 488 was purchased from Invitrogen, Life technology Europe, Bleiswijk, Netherlands. Primary dermal fibroblasts: normal, human, and adult cells were purchased from ATCC, Manassas, VA, USA. Human Colo 818 (malignant melanoma) cells were bought from DSMZ, Braunschweig Germany. The Dulbecco’s Modified Eagle Medium (DMEM) [supplemented with 1% (*v/v*) penicillin/streptomycin, 2% (*v/v*) L-glutamate, and 10% (*v/v*) fetal bovine serum (FBS)], penicillin/ streptomycin, phosphate-buffered saline (PBS), and L-glutamate were obtained from Sigma-Aldrich, Darmstadt, Germany.

### 2.2. Synthesis of Periodic Mesoporous Organosilica (PMO-PFC)

Briefly, 30 mL ethanol (99.8%) and 90 mL deionized (DI) water were mixed by magnetic stirrer in a 250 mL round-bottom flask. While stirring, 485 mg CTAB and 270 μL NH_3_ (32%) were added and stirred for 1 h at room temperature (RT). Then, 1.74 mL (1.67 g, 4.7 m mol) BTEE and PFC (0.59 g, 1.16 m mol, 0.44 mL in 3 mL ethanol) were added and stirred for additional 48 h at RT. After 48 h and while still being stirred, 50 mL ethanol (99%) was added, followed by 1.4 mL HCl (32 wt%) which was slowly added to the mixture and stirred for 6 h at 50 °C to remove the CTAB. The reaction mixture was transferred to 15 mL falcon tubes and centrifuged (6500 rpm, Hettich EBA 200 small centrifuge from Andreas Hettich GmbH & Co.KG, Tuttlingen, Germany) for 15 min at RT. The supernatant was discarded and the PMO-PFC particles were washed a further 3 times with ethanol by centrifugation. The tubes with particles were left with open lids to dry for 2 days at RT.

### 2.3. Loading of Rutin to PMO-PFC Particles

An amount of 100 mg of PMO-PFC particles and rutin trihydrate (80 mg) were mixed together with 0.4 mL ethanol, sonicated for 20 min and stirred for 10 min at RT. Then, 1.1 mL DI water was added and sonicated again for 5 min. The mixture was stirred overnight at RT and then centrifuged for 15 min to collect the rutin-coated PMO-PFC particles. The coated particles were further washed with DI water and dried overnight. The supernatant was collected for the purpose of measuring rutin concentration. The amount of rutin loaded into the PMO-PFC particles was determined by means of spectrophotometric analyses. 300 μL of supernatant was added to a 96-well plate and the absorbance measured at 352 nm by a UV-vis spectrometer. The concentration of free rutin was calculated by using a calibration curve (Figure S1). The amount of loaded rutin was the amount of rutin added initially minus the amount of free rutin in the supernatant. The loaded efficiency (E%) was calculated according to the equation below:

E% = (amount of rutin added-amount of free rutin)/(amount of rutin added) × 100

### 2.4. Coating of Poly-d-lysine (PDL) to Ru(PMO-PFC) Particles

An amount of 50 mg of Ru(PMO-PFC) was added to 1.5 mL PDL solution (0.5 mg/mL in DI water) in a 2 mL Eppendorf tube, sonicated for 20 min, and then stirred for 1 day at RT. The mixture was then centrifuged for 15 min and the supernatant was used to calculate the released rutin by means of spectrophotometric analyses. The absorbance and E% were measured as explained in the above section and supporting information. The product was dried, lids open, at RT.

### 2.5. Preparation of GelMa

Gelatin methacryloyl (GelMa) was synthesized according to the previous study [44]. First, gelatin (20 g) was dissolved in PBS (600 mL) at 60 °C for 1 h by stirring. Then, methacrylic anhydride (16 mL) was added dropwise to the mixture and continuously stirred for 2 h at 50 °C. The entire mixture was then transferred into dialysis membrane tubes (with 12–14 kDa) and kept in autoclaved DI water for 7 days at 50 °C to remove unreacted methacrylic anhydride. The water was replaced every day with fresh prewarmed and autoclaved DI water. After 7 days, the solution was filtered with a vacuum filter (110 mm pore size) and then lyophilized to achieve a dried GelMa foam.

### 2.6. Preparation of NC Hydrogel and Scaffold

A GelMa stock solution was first prepared with GelMa (1 g, 10 *w/v*%) in PBS (10 mL) mixed with eosin Y in PBS (0.1 mM) as a photoinitiator, TEA as a co-initiator (133 µL, 1.3 *w/v*%), and VC as a co-monomer (0.1 g, 1.0 *w/v*%) and kept at 80 °C for 10 min in order to dissolve GelMa. For the scaffold, the stock GelMa solution was mixed with particle (1 mg/mL) and alginate (70 mg/mL). The hydrogel solution was homogeneously mixed by spatula and the mixed solution was transferred into a syringe. The hydrogel solution was then filled into a hexagon template (side—0.25 cm, height—0.4 cm, volume = ca. 65 µL) by syringe and photo-crosslinked with visible light (450–550 nm) for 120–180 s, using FocalSeal (Genzyme Biosurgical, Cambridge, MA, USA). For ionic crosslinking of Alg, the templates were covered with 2.5 mM CaCl_2_ solution in DI water for 20 min. For rheology, cross-linked hydrogel was removed from the templates and used for the measurement. For scaffolds, the templates were frozen at −20 °C for 1 day and then lyophilized with a freeze dryer. The hexagon template was removed and the scaffolds were ready to use for experimentation. For mechanical properties, the cylindrical scaffolds (10 mm diameter, 10–13 mm height) were freeze dried and prepared similarly to the above-mentioned method.

### 2.7. Rutin Release from Rutin-Coated GelMa-Based Scaffold

First, the rutin-coated GelMa-based scaffold (freeze-dried) was placed in DMEM (1 mL, pH-6 and 7.4) in a 2 mL Eppendorf tube at RT. After each incubation time, the media was collected and centrifuged for 10 min. The supernatant was transferred into a new Eppendorf tube and fresh media was added to the scaffold and left at RT until the next incubation time (adding up the previous incubation time). The collected supernatants were used to measure the absorbance of rutin for each incubation time by spectrophotometric analyses. The concentration of released-rutin was calculated by using a calibration curve similar to the method discussed in the supporting information (Figure S1).

### 2.8. Measurement of O_2_ Content from Scaffold

The O_2_ content of the cell culture media and scaffold containing cell culture media was determined using an oxygen sensor (OXY-1 SMA trace, Pre Sens Precision Sensing GmbH, Regensburg, Germany). All samples were incubated either in normoxia (21% O_2_) or hypoxia conditions (1% O_2_) for 14 days. To generate the hypoxia condition, we used a hypoxia box saturated with a gas mixture of 1% O_2_ + 5% CO_2_ + 94% N_2_. First, the scaffold-containing media was placed in a hypoxia box closed with a rubber cap and saturated with 1% O_2_, and the oxygen level measured after the given incubation time. Similar was done for the normoxia condition without saturating with 1% O_2_.

### 2.9. 3D Printing of Hydrogels

To show the printability of prepared NC hydrogels, NC hydrogels were prepared with particles (1 mg/mL) and alginate (70 mg/mL). The hydrogel mixture was homogenously mixed by spatula and transferred into a special syringe for 3D printing. The hexagon structure (ca. 2.3 × 2.3 cm) was designed with the inbuilt software of a Cellink 3D printer (CELLINK, Boston, MA, USA) for layer-by-layer deposition (3 layers). Particles containing hydrogels were printed (using Cellink HeartWare version 2.4.1) in a hexagon structure, and crosslinked with visible light (450–550 nm) for 120–180 s using FocalSeal (Genzyme Biosurgical, Cambridge, MA, USA), for covalent photo-crosslinking of GelMA, and then with a 2.5 mM CaCl_2_ solution for ionic crosslinking of Alg. The syringe temperature and printing plate temperature were set at 37 °C. A needle with an inner diameter of 0.41 mm was used for printing, and the speed of the syringe was 20 mm s^−1^. To prepare the scaffolds, the samples were frozen at −20 °C and then lyophilized with a freeze dryer, yielding the 3D-printed scaffolds.

### 2.10. Cell Viability in the Scaffolds

First, PMO-PFC, Ru(PMO-PFC), and Ru(PMO-PFC)PDL particles(1 mg/mL) -prepared scaffolds were placed on a culture plate. The cells were thawed from −80 °C and carefully seeded on the culture plate with pre-warmed media. After overnight incubation at 37 °C and 5% CO_2_, cells were washed with PBS and harvested by trypsinization. Then, cells were collected by adding pre-warmed media and centrifuged for 3 min. The supernatant was discarded and the cell pallet was collected by adding 1 mL pre-warmed media. Cells were counted in hemocytometer and the particle-coated scaffolds were incubated with 10^4^ cells for 1 day and 7 days under hypoxia and normoxia conditions at 37 °C and 5% CO_2_. For control, the cells were seeded on scaffold without particles (only the GelMa-based scaffold). After incubation period, the scaffolds with cells were washed with PBS (2×) and incubated with cell proliferation reagent WST-1 assay (10 vol% in media) for 3 h. Cell viability was measured at 460 nm by scanning the plate with a UV-vis spectrophotometer.

### 2.11. Co-Staining of Cells

Cells were seeded separately on the scaffolds and incubated for 1 day and 7 days at 37 °C and 5% CO_2_ under hypoxia and normoxia conditions. Then, the cells were fixed with paraformaldehyde (4%). After 20 min, scaffolds were washed with PBS (2×) and the nuclei-staining was performed with Hoechst 33342 dye [stock solution (16.2 mM), diluted 1:2000 in PBS] at RT for 20 min. Then, scaffolds were washed with PBS (2×) and incubated with 0.1% Triton X-100 in PBS for 30 min at RT. Scaffolds were then washed with PBS (3×), and co-stained with phalloidin Alexa Fluor 488 [5 µL of the methanolic stock solution (6.6 µM) of phalloidin Alexa Fluor 488, diluted in 200 µL of PBS containing 3% bovine serum albumin] for f-actin staining. Scaffolds were kept overnight at room temperature and stored in the dark. Afterward, the scaffolds were washed with PBS (2×) and ready for imaging.

### 2.12. Characterization

Scanning electron microscopy (SEM) was performed on a Zeiss cross beam 340 scanning electron microscope, to determine the morphology of the particles. The average size of the particles from SEM images was measured by ImageJ from 35 NPs. An InlensDuo detector (secondary electron (SE) detector) for particles and a SESI detector (SE, and secondary ion detector) for the hydrogels were used. Zeta potential measurements and dynamic light scattering (DLS) were conducted using a Malvern Zetasizer Nano Series. A Nikon ECLIPSE Ts2R fluorescence microscope was used to determine the cell morphology and fluorescence imaging. Cell viability was measured using a Tecan Infinite 200 PRO spectrophotometer. A Christ Alpha 1-2-LD plus freeze-dryer was used to produce porous hydrogel scaffolds. An INKREDIBLE 3D bioprinter (CELLINK, Boston, MA, USA) was used to print all nanocomposite hydrogels into computer-designed 3D structures. Rheological measurements were carried out using an MCR 302 rheometer (Anton Paar, Ashland, VA, USA) with a 25 mm diameter parallel-plate geometry measuring system. A material testing machine (type 066590, ZwickRoell GmbH & Co. KG, Ulm, Germany) was used to determine the compression modulus of the samples.

### 2.13. Statistical Methods

Experiments were performed three times. The results are shown as average values with standard deviations. Significance tests were conducted using a single factor ANOVA test. Significance levels were depicted as * for *p* ≤ 0.05, ** for *p* ≤ 0.01, and *** for *p* ≤ 0.001, where *p* is the probability value and a statistical measurement used to validate a hypothesis against observed data.

## 3. Results

### 3.1. Preparation and Characterization of Rutin-Coated PMO-PFC Particles

**Figure 1:**
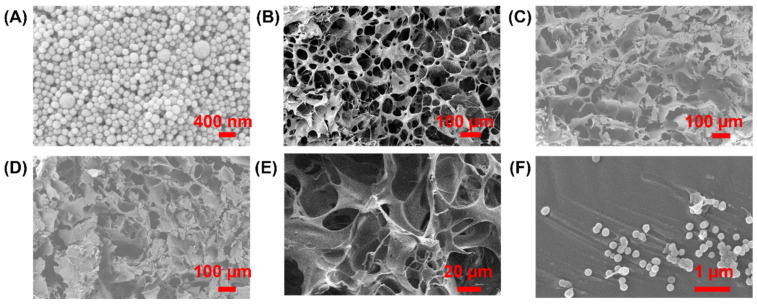
SEM images of PMO-PFCs (**A**), G+PMO-PFC (**B**), G+Ru(PMO-PFC) (**C**), G+Ru(PMO-PFC)PDL (**D**), zoom-in image of hydrogel (**E**) and PMO-PFCs inside the hydrogel network (**F**).

The synthesis of PMO-PFC particle was performed according to a previous study [41]. The PFCs are excellent candidates for O_2_-releasing materials, but the hydrophobic and non-aqueous nature of PFCs can exert a sudden release of O_2_ [45]. Therefore, the internal and external surfaces of NPs were functionalized with PFCs. Next, rutin was encapsulated into the PMO-PFC system (denoted as Ru(PMO-PFC)) as drug molecules via hydrophobic forces generated by PFC. It is important to note that non-PFC coated periodic mesoporous organosilica is insufficient for hydrophobic rutin encapsulation. Rutin is a naturally occurring bioflavonoid and has a wide range of pharmacological properties. The poor water solubility of rutin limits its bioavailability; researchers have, therefore, incorporated rutin into the nanosystems to enhance their stability and bioavailability. We calculated around 90% loading efficiency of rutin into PMO-PFCs. The hydrophobic nature of rutin allows entrapment into PMO-PFC particles by hydrophobic forces [46]. The calibration curve of rutin in water and calculations are given in the Supplementary Information (Figure S1). Further, to enhance the stability of the whole system in biological and physiochemical conditions, a biodegradable polymer, PDL, was coated over the rutin-functionalized PMO-PFCs, denoted as Ru(PMO-PFC)PDL. During the PDL-coating process, an amount of rutin is expected to release into the supernatant. Therefore, we calculated around 83% loading efficiency after the PDL coating into the rutin-encapsulated PMO-PFC particles. The characterization of the functionalized nanoparticles was performed by scanning electron microscopy (SEM), dynamic light scattering (DLS), and zeta potential measurement. The measured zeta potential and size distribution values are shown in Table 1. The changes in the zeta potential from −6.98 ± 0.28 to −14.94 ± 0.47 mV indicate a combined deposition/adsorption process of rutin on the surface of the PMO-PFCs, because rutin displays different solubility in water at different temperatures that is attributed to the different interaction forces such as van der Waals forces and hydrogen bonding (Peng et al.) [47]. The deposition/adsorption of rutin on the surface of PMO-PFCs, increases the value of the zeta potential; the negative value may be due to its hydroxyl groups, which indicates the whole particles now have more negative zeta potential. This result also indicates the deposition/adsorption of rutin on the external surface of PMO-PFC. Further, the size of the particles increases from 238.30 ± 32.18 to 457.60 ± 60.89 nm. After the PDL (positively charged polymer)-coating of Ru(PMO-PFC), a positive increase in the zeta potential indicates the successful coating of the polymer matrix on the Ru(PMO-PFC) via electrostatic interaction. The morphology of the nanoparticles is shown in SEM image (Figure 1A). The average size of the particles in DLS and SEM is different, which is most likely due to the aggregation of the hydrophobic particles in the aqueous solution during the DLS measurement. Furthermore, the increase in the size of the Ru(PMO-PFC)PDL can be due to the swelling of the PDL on the surface of the particles.

### 3.2. Characterization of NC Hydrogels

After successful functionalization of rutin to PMO-PFCs and PDL coating on Ru(PMO-PFC), the hydrogel precursor and scaffolds were prepared as described in the Method section. The scaffolds with PMO-PFC, Ru(PMO-PFC) and Ru(PMO-PFC)PDL are denoted as G+PMO-PFC, G+Ru(PMO-PFC), G+Ru(PMO-PFC)PDL, respectively. The hydrogels were also characterized for morphological properties by SEM (Figure 1B–E). The SEM images show the porous structure of the prepared scaffolds. The average size of the PMO-PFCs is 143 ± 12 nm (Figure 1A). The zoom-in image (Figure 1F) shows the distribution of PMO-PFCs within hydrogel network. The aggregation of PMO-PFCs is expected, due to the hydrophobic nature of the particles.

**Figure 2:**
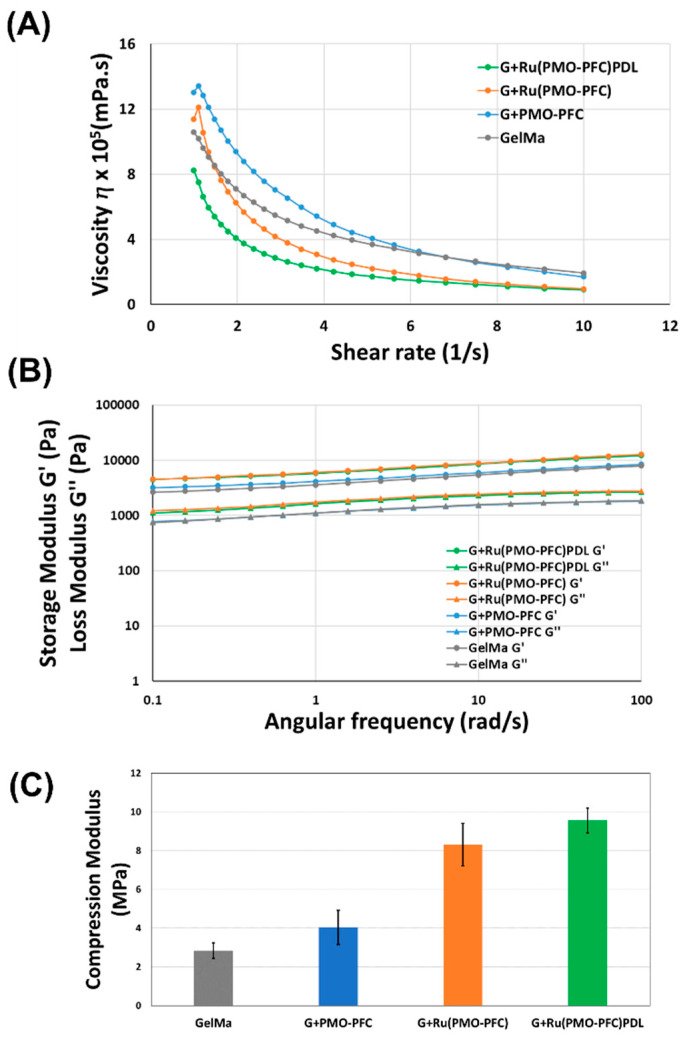
Rheological and mechanical properties of NC hydrogels. Viscosity vs shear rate (**A**), the storage modulus is shown by circle and loss modulus by triangle marker vs angular frequency (**B**), and compression modulus for each hydrogel scaffold (**C**).

Rheologic properties such as viscosity (η), storage (G′) and loss modulus (G″), are important factors to determine the injectability of the hydrogel. The ability of hydrogels to change viscosity in response to the changes in shear stress is crucial for injectable hydrogels. Hydrogels with a lower viscosity and lower storage and loss moduli are typically easier to inject than hydrogels with a high viscosity and high storage and loss moduli. The viscosity of the G+PMO-PFC, G+Ru(PMO-PFC) and G+Ru(PMO-PFC)PDL hydrogels decreases with increasing shear rate and demonstrates the shear thinning property (Figure 2A). Storage and loss moduli are the elastic and viscous responses of a hydrogel to oscillatory shear. A higher storage modulus than loss modulus for the G+PMO-PFC, G+Ru(PMO-PFC) and G+Ru(PMO-PFC)PDL hydrogels over the entire range of angular velocities demonstrates their viscoelastic properties (Figure 2B). The shear thinning and viscoelastic properties of prepared NC hydrogels show their ability for use as injectable hydrogels and as hydrogel precursors for printing 3D scaffolds. For the compression test, the cylindrical scaffolds were prepared according to the earlier-mentioned method. The NC-hydrogel scaffolds show a higher compression modulus than the GelMa-based scaffold at 100 N (Figure 2C), indicating stronger mechanical compressive properties of NC hydrogel, due to incorporation of NPs. This is due to the non-covalent interactions between the polymer networks of hydrogel- and O_2_-carrying NPs. The G+Ru(PMO-PFC) shows a higher compression modulus than the GelMa-based scaffold and G+PMO-PFC, perhaps because of the interaction of GelMa with the hydroxyl-groups of the rutin. G+Ru(PMO-PFC)PDL shows more stiffness (highest compression modulus among others) due to the distribution of hydrophilic PDL-coated particles within the hydrogel network. The swelling and degradation of the scaffold were also measured (Figures S2 and S3). The G+PMO-PFC, G+Ru(PMO-PFC), and G+Ru(PMO-PFC)PDL scaffolds exhibited a low swelling ratio and less degradation compared with the GelMa-based scaffold (see Supplementary Information). To summarize, the prepared NC-hydrogel scaffolds show better rheological properties which enables them to be used as injectable and printable hydrogels. The enhanced mechanical properties also make these NC hydrogels potential candidates for constructing an artificial tissue construct.

### 3.3. 3D Printing of NC Hydrogels

**Figure 3:**
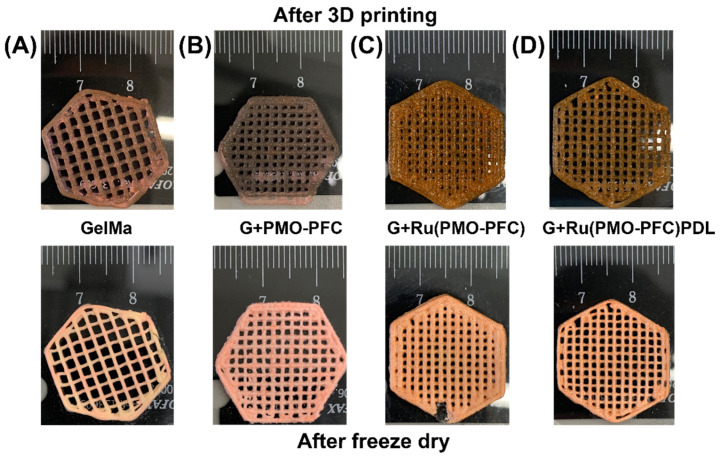
Photograph of 3D printed scaffolds of GelMa (**A**), G+PMO-PFC (**B**), G+Ru(PMO-PFC) (**C**) and G+Ru(PMO-PFC)PDL (**D**) (The top images are after printing; bottom images are after crosslinking and freeze-drying process).

A 3D-printable hydrogel is an emerging area for tissue engineering because of the bioavailability and biodegradability of hydrogels, and their capabilities for cell adhesion, proliferation and differentiation. The improved mechanical and elastic properties of NC hydrogels make them suitable candidates for the strong and durable artificial organ implantations [48]. To demonstrate the printability of the prepared NC hydrogels, NC hydrogels were printed into a hexagonal structure using the Cellink 3D printer. Figure 3 shows the 3D hexagons printed in this way (Figure 3, top images). The crosslinked and freeze-dried scaffolds retained their shape fidelity (Figure 3, lower images).

### 3.4. Oxygen Release from NC Hydrogel Scaffold

**Figure 4:**
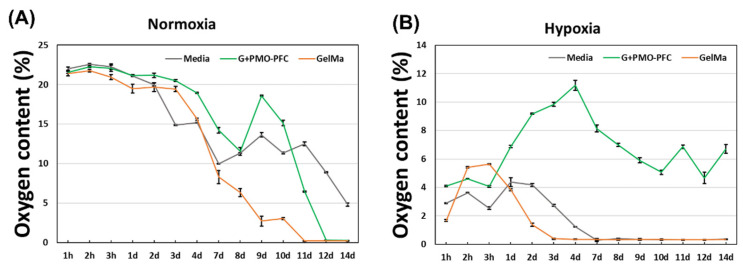
O_2_ content of NC hydrogel scaffold under normoxia (**A**) and hypoxia condition (**B**).

To test the oxygen-carrying capacity of PMO-PFC-containing scaffolds, the scaffolds were placed in Dulbecco’s Modified Eagle Medium (DMEM) and their O_2_ content was measured by an oxygen sensor under normoxia and hypoxia conditions. The scaffold-containing media was kept in a hypoxia box at normoxia (Figure 4A) and hypoxia (Figure 4B) conditions for given incubation times. Under the hypoxia condition, O_2_ content increases for the media with G+PMO-PFC and remains constant or higher than others for the longer period (Figure 4B), while that of the particle-free scaffold (GelMa, control) decreases after 3 h and shows almost zero O_2_ content over the longer period. A similar trend is observed with media without any scaffold. However, the media with G+PMO-PFC in normoxia conditions shows constant O_2_ content over the period of 4 days and then starts decreasing after 4 days. However, the decrease in O_2_ content is slower in G+PMO-PFC than that in the GelMa-based scaffold (particle-free scaffold). For G+PMO-PFC, the oxygen-carrying nanoparticles release O_2_ under hypoxia and normoxia conditions. It is important to note that under normoxia conditions, the scaffolds in the media interact with air, while under hypoxia they are in a closed hypoxia box at about 1% O_2_. Therefore, the release of O_2_ from PMO-PFC-containing scaffolds was different under normoxia and hypoxia conditions. To summarize, scaffolds containing PMO-PFC particles continuously release oxygen in the hypoxia condition and maintain oxygen levels in normoxia conditions (at least for 4 days) as compared with particle-free scaffolds.

### 3.5. Rutin Release from Rutin-Functionalized NC Hydrogel Scaffold

**Figure 5:**
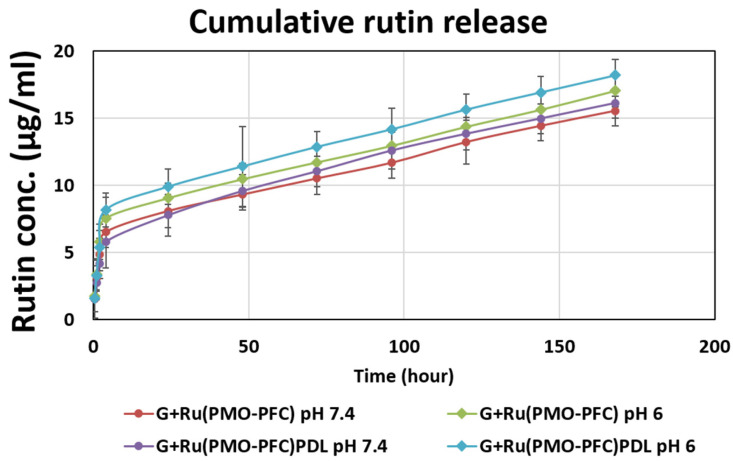
Rutin release (µg/mL) from NC-hydrogel scaffolds at pH 7.4 and pH 6.

Rutin release from the NC scaffolds was studied to show the ability of bio-functional PMO-PFC particles for sustained drug release into the hydrogel. The initial amount of rutin per G+Ru(PMO-PFC) scaffold was ca. 46.7 µg and 42.3 µg for the G+Ru(PMO-PFC)PDL scaffold (see the supporting information for the calculation). The rutin release was performed at pH 7.4 (physiological environment) and pH 6 (tumor cell environment). Briefly, the G+Ru(PMO-PFC) and G+Ru(PMO-PFC)PDL scaffolds were placed in media for the given incubation time (30 min to 7 days) at room temperature. After reach incubation time, the media was replaced by fresh media and the absorbance of collected media was measured by UV spectroscopy. The concentration of rutin into media was then calculated from the calibration curve. The cumulative release profile (Figure 5) shows sustained release for pH 7.4 and pH 6. It is observed that at pH 6 the release of rutin is slightly higher than the release at pH 7.4 for both scaffolds. The percentage graph is shown in Figure S4. The slower and constant drug released from the hydrogels could be beneficial at a specific site until the natural tissue regenerates or is replaced by implanted artificial tissue. The slightly higher release at pH 6 could be beneficial, for example, for anticancer drug release at the tumor site. The results show that the release of rutin from NC hydrogels is sustained but not significantly pH dependent.

### 3.6. Cell-Viability Experiment on NC-Hydrogel Scaffolds

**Figure 6:**
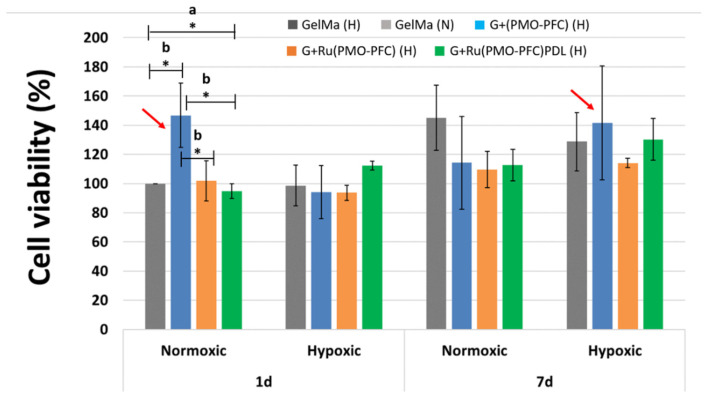
Cell viability of FB cells under normoxia and hypoxia conditions for 1 day and 7 days incubation times. The red arrow shows the sample with the highest effect on cell viability. ANOVA: *p* < 0.05 (*), a = significant difference between four groups, b = significant difference between two groups. Number of repeated experiments (N) = 3.

To test the bio-functionality and cell-adhesiveness of the prepared NC-hydrogel scaffolds, fibroblasts cells (FBs) were used. FBs are the most common cell type present in connective tissue and play an important role in tissue repair and wound healing. For the cell experiment, the scaffolds were placed into the well plate and cells (10^4^) were incubated for 1 day and 7 days, at 37 °C and 5% CO_2_. The cell proliferation reagent WST-1 was used for the spectrophotometric quantification of cell viability. Figure 6 shows the cell experiment data for cell viability versus each sample for the number of incubation days. The data were normalized to the control sample (GelMa, 1day under normoxia) for each condition and incubation time. Hypoxia conditions occur when there is insufficient (lower than normal oxygen level) and heterogeneous oxygen distribution within the body tissues, which can reduce cell growth and proliferation. Hypoxia can reduce cell apoptosis, tissue necrosis, transplantation failure, and failure in tissue formation. However, oxygen-carrying particles within NC-hydrogel scaffolds can release oxygen and increase proliferation.

Our results show (Figure 6) that, at one day normoxia, no significant difference in the number of viable cells was observed for G+Ru(PMO-PFC) and G+Ru(PMO-PFC)PDL compared to GelMa-based scaffolds, while G+(PMO-PFC) exhibited approximately 47% more cell viability than GelMa, demonstrating the positive impact of O_2_-carrying PMO-PFC on enhanced cell viability under normoxic conditions. However, under hypoxia conditions, after one day of incubation, we observed only an enhanced effect of G+Ru(PMO-PFC)PDL on cell viability (approximately 14% increase in cell viability). On the other hand, cell viability increased under normoxia from one day to seven days by approximately 45, 7, and 19% for GelMa, G+Ru(PMO-PFC) and G+Ru(PMO-PFC)PDL, respectively. However, these results were not significant for GelMa, so the effects of releasing O_2_ and/or rutin on cell viability after seven days of incubation under normoxia are not apparent. Under hypoxia conditions, after seven days of incubation, cell viability was slightly lower than under normoxia only for GelMa. However, we observed a slight increase in cell viability in G+(PMO-PFC), G+Ru(PMO-PFC) and G+Ru(PMO-PFC)PDL, respectively. Furthermore, cell viability from one day to seven days also showed an increase in cell viability under hypoxia conditions due to sustained O_2_ release, the antioxidant effect of rutin suppressing oxidative stress induced by hypoxia [23], and the cell-adhesive GelMa and PDL coating.

**Figure 7:**
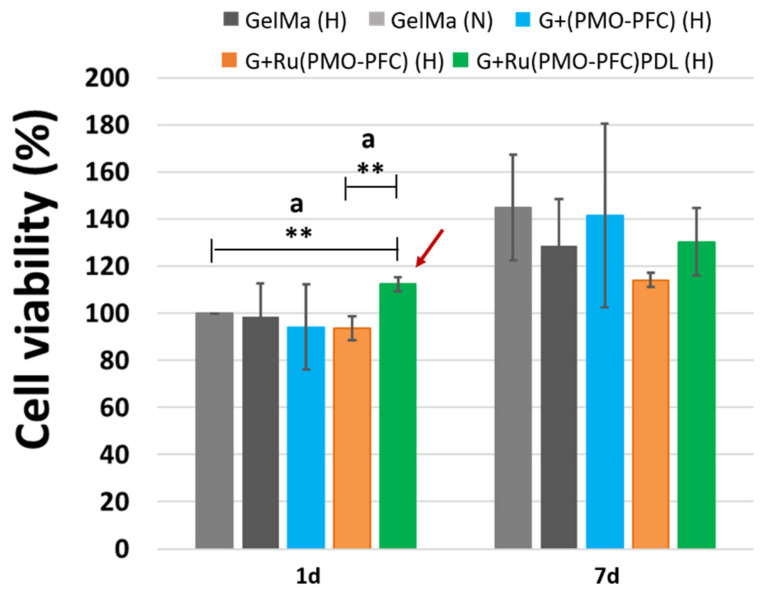
Comparison of the effects of G+(PMO-PFC), G+Ru(PMO-PFC), and G+Ru(PMO-PFC)PDL on cell viability under hypoxia conditions with the effects of the particle-free GelMa-based scaffold under normoxia conditions (H = hypoxia; N = normoxia). The dark red arrow shows the sample with the highest effect on cell viability. ANOVA: *p* < 0.01 (**), a = significant difference between two groups. Number of repeated experiments (N) = 3.

In general, our results for one day hypoxia, and seven days’ normoxia and hypoxia showed no significant differences (Figure 6); we, therefore, compared the effects of G+(PMO-PFC), G+Ru(PMO-PFC), and G+Ru(PMO-PFC)PDL on cell viability under hypoxia conditions with the effects of the particle-free GelMa-based scaffold under normoxia conditions (Figure 7). We aimed, in fact, to maintain cell viability under hypoxia using O_2_ delivery particles similar to cell viability under normoxia conditions. We observed that incorporation of only G+Ru(PMO-PFC)PDL (H) into GelMa was able to increase cell viability under hypoxia (H) conditions more than cell viability in GelMa under normoxia (N) conditions for a one-day incubation period. While GelMa (H), G+(PMO-PFC) (H), and G+Ru(PMO-PFC) (H) showed approximately 2%, 6%, and 7% less cell viability than GelMa (N), G+Ru(PMO-PFC)PDL (H) showed a 12% higher cell viability than GelMa (N). This result indicates the synergistic positive effect of O_2_, rutin and PDL on cell viability under hypoxia conditions. After seven days of incubation, all samples displayed less cell viability under hypoxia than normoxia conditions. However, G+(PMO-PFC) (H) showed the best result compared with the other samples. Cell viability for GelMa (H), G+Ru(PMO-PFC) (H), and G+Ru(PMO-PFC)PDL (H) was 11, 21, and 10% less than GelMa (N), respectively, while cell viability for G+(PMO-PFC) (H) was only 2% less than GelMa (N). These results indicate that G+Ru(PMO-PFC)PDL and G+(PMO-PFC) can be used for one- and seven-day incubation periods, respectively, to improve or support cell viability under hypoxia conditions. Furthermore, their impact on cell viability increased over time, showing that they can support viability of cells for longer incubation times.

To summarize, the scaffold containing G+(PMO-PFC) was the best sample that increased cell viability under normoxia only after one day of incubation (Figure 6, red arrow), while it supported cell viability under hypoxia similar to normoxia but only after seven days of incubation (Figure 7, red arrow). On the other hand, G+Ru(PMO-PFC)PDL was the best sample that allowed us to increase cell viability under hypoxia slightly more than under normoxia conditions, although this effect was significant only after one day of incubation (Figure 7, dark red arrow).

**Figure 8:**
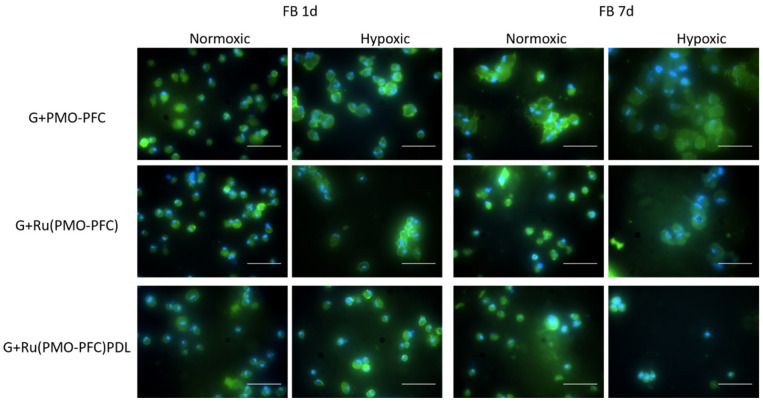
Fluorescence images of FB cells on NC-hydrogel scaffolds under normoxia and hypoxia conditions for 1d and 7d incubation times. Scale bar equals 50 µm.

Fluorescence microscopy was used to examine the morphology of the cells on the samples (Figure 8). For this, cells were incubated on NC-hydrogel scaffolds under normoxia and hypoxia for 1 day to 7 days. After incubation period, cells were nuclei- stained with Hoechst 33342 dye and actin-stained with phalloidin Alexa Fluor 488 dye. Degraded scaffolds with FB cells on the culture plate were then imaged by microscope. We observed that the FB cells on the scaffolds had a circular shape instead of their usual elongated and stretched shape. The ECM nature of hydrogels helps the cells to grow in different shapes and the shapes of the cells can be manipulated by biochemical clues and the mechanical properties of the scaffolds [49,50].

## 4. Conclusions

We successfully described a method for the fabrication of NC hydrogels for co-administration of hydrophobic drug molecules and O_2_ delivery. NC hydrogels provide sustained O_2_ content over a 14-day period under hypoxia and normoxia conditions. The release experiment also suggests the sustained drug release for seven days. The beneficial effects of nontoxic and antioxidant rutin, cell-adhesive coating of PMO-PFCs in the 3D hydrogel networks, and O_2_ release from the scaffolds on cell viability were observed only for G+(PMO-PFC and G+Ru(PMO-PFC)PDL under specific conditions and incubation times. G+(PMO-PFC) promoted cell viability under normoxia only after one day of incubation and under hypoxia after seven days of incubation. G+Ru(PMO-PFC)PDL increased cell viability more under hypoxia than under normoxia conditions in one day of incubation, demonstrating the synergistic effect of O_2_ release, rutin, and PDL on cell viability. However, our results show that our systems still need to be improved to achieve better cell viability under hypoxia conditions. The improved rheological properties suggest the prepared NC hydrogels can be further tested or used as an injectable hydrogel. The enhanced mechanical properties and 3D-printability of NC hydrogels may be used for artificial tissue constructs. Therefore, overall performance of prepared NC hydrogels makes an alternate attractive route for injectable hydrogel in local area delivery and tissue regeneration for implantation. This can be further investigated for wound dressing to test the pharmacological properties of rutin for in vivo application.

## Figures and Tables

**Table 1:** Zeta potential (mean value ± standard deviation) and size measurement (mean value ± standard deviation) of particles. ANOVA: *p* < 0.01 (**), *p* < 0.001 (***) and ^a^ = significant difference between PMO-PFC and Ru(PMO-PFC), ^b^ = significant difference between Ru(PMO-PFC) and Ru(PMO-PFC)PDL. Number of repeated experiments (N) = 3.

Sample	Zeta Potential (mV)	Size (nm)
PMO-PFC	−6.98 ± 0.28	238.30 ± 32.18
Ru(PMO-PFC) ^a^	−14.94 ± 0.47 ***	457.60 ± 60.89 **
Ru(PMO-PFC)PDL ^b^	44.50 ± 1.52 ***	520.76 ± 20.11 ***

## Data Availability

Not applicable.

## Supplementary Materials

The following supporting information can be downloaded at: https://www.mdpi.com/article/10.3390/nano12081304/s1, Figure S1: Calibration curve of rutin in water at wavelength of 352 nm (A). The absorbance spectra of supernatant after rutin coating of PMO-PFCs (B); Figure S2: Swelling ratio of GelMa and NC hydrogels at 1day and 7 days; Figure S3: Degradation of GelMa and NC hydrogels at 1 day and 7 days; Figure S4: Rutin release in percentage for scaffolds at different pH value.

## References

[1] Gaharwar A.K., Peppas N.A., Khademhosseini A. (2014). Nanocomposite Hydrogels for Biomedical Applications. Biotechnol. Bioeng..

[2] Rafieian S., Mirzadeh H., Mahdavi H., Masoumi M.E. (2019). A review on nanocomposite hydrogels and their biomedical applications. Sci. Eng. Compos. Mater..

[3] Lavrador P., Esteves M.R., Gaspar V.M., Mano J.F. (2020). Stimuli-Responsive Nanocomposite Hydrogels for Biomedical Applications. Adv. Funct. Mater..

[4] Huang J., Liu F., Su H., Xiong J., Yang L., Xia J., Liang Y. (2022). Advanced Nanocomposite Hydrogels for Cartilage Tissue Engineering. Gels.

[5] Lee J.H. (2018). Injectable hydrogels delivering therapeutic agents for disease treatment and tissue engineering. Biomater. Res..

[6] Nichol J.W., Koshy S.T., Bae H., Hwang C.M., Yamanlar S., Khademhosseini A. (2010). Cell-laden microengineered gelatin methacrylate hydrogels. Biomaterials.

[7] Song F., Li X., Wang Q., Liao L., Zhang C. (2015). Nanocomposite Hydrogels and Their Applications in Drug Delivery and Tissue Engineering. J. Biomed. Nanotechnol..

[8] Rizzo F., Kehr N.S. (2020). Recent Advances in Injectable Hydrogels for Controlled and Local Drug Delivery. Adv. Healthc. Mater..

[9] Tutar R., Motealleh A., Khademhosseini A., Kehr N.S. (2019). Functional Nanomaterials on 2D Surfaces and in 3D Nanocomposite Hydrogels for Biomedical Applications. Adv. Funct. Mater..

[10] Hou L., Shi Y., Jiang G., Liu W., Han H., Feng Q., Ren J., Yuan Y., Wang Y., Shi J. (2016). Smart nanocomposite hydrogels based on azo crosslinked graphene oxide for oral colon-specific drug delivery. Nanotechnology.

[11] Pacelli S., Maloney R., Chakravarti A.R., Whitlow J., Basu S., Modaresi S., Gehrke S., Paul A. (2017). Controlling Adult Stem Cell Behavior Using Nanodiamond-Reinforced Hydrogel: Implication in Bone Regeneration Therapy. Sci. Rep..

[12] Zhao W., Odelius K., Edlund U., Zhao C., Albertsson A.-C. (2015). In Situ Synthesis of Magnetic Field-Responsive Hemicellulose Hydrogels for Drug Delivery. Biomacromolecules.

[13] Alvarez G.S., Hélary C., Mebert A.M., Wang X., Coradin T., Desimone M.F. (2014). Antibiotic-loaded silica nanoparticle–collagen composite hydrogels with prolonged antimicrobial activity for wound infection prevention. J. Mater. Chem. B.

[14] Motealleh A., Kart D., Czieborowski M., Kehr N.S. (2021). Functional Nanomaterials and 3D-Printable Nanocomposite Hydrogels for Enhanced Cell Proliferation and for the Reduction of Bacterial Biofilm Formation. ACS Appl. Mater. Interfaces.

[15] Motealleh A., Dorri P., Kehr N.S. (2020). Injectable polymer/nanomaterial composites for the fabrication of three-dimensional biomaterial scaffolds. Biomed. Mater..

[16] Zhou L., Chen F., Hou Z., Chen Y., Luo X. (2021). Injectable self-healing CuS nanoparticle complex hydrogels with antibacterial, anti-cancer, and wound healing properties. Chem. Eng. J..

[17] Dimatteo R., Darling N.J., Segura T. (2018). In situ forming injectable hydrogels for drug delivery and wound repair. Adv. Drug Deliv. Rev..

[18] Spicer C.D. (2020). Hydrogel scaffolds for tissue engineering: The importance of polymer choice. Polym. Chem..

[19] Nezhad-Mokhtari P., Ghorbani M., Roshangar L., Soleimani Rad J. (2019). A review on the construction of hydrogel scaffolds by various chemically techniques for tissue engineering. Eur. Polym. J..

[20] El-Sherbiny I.M., Yacoub M.H. (2013). Hydrogel scaffolds for tissue engineering: Progress and challenges. Glob. Cardiol. Sci. Pract..

[21] Gullón B., Lú-Chau T.A., Moreira M.T., Lema J.M., Eibes G. (2017). Rutin: A review on extraction, identification and purification methods, biological activities and approaches to enhance its bioavailability. Trends Food Sci. Technol..

[22] Ganeshpurkar A., Saluja A.K. (2017). The Pharmacological Potential of Rutin. Saudi Pharm. J..

[23] Negahdari R., Bohlouli S., Sharifi S., Maleki Dizaj S., Rahbar Saadat Y., Khezri K., Jafari S., Ahmadian E., Gorbani Jahandizi N., Raeesi S. (2020). Therapeutic benefits of rutin and its nanoformulations. Phytother. Res..

[24] Júlio A., Caparica R., Costa Lima S.A., Fernandes A.S., Rosado C., Prazeres D.M.F., Reis S., Santos de Almeida T., Fonte P. (2019). Ionic Liquid-Polymer Nanoparticle Hybrid Systems as New Tools to Deliver Poorly Soluble Drugs. Nanomaterials.

[25] Wu H., Su M., Jin H., Li X., Wang P., Chen J., Chen J. (2020). Rutin-Loaded Silver Nanoparticles With Antithrombotic Function. Front. Bioeng. Biotechnol..

[26] Hu B., Dai F., Fan Z., Ma G., Tang Q., Zhang X. (2015). Nanotheranostics: Congo Red/Rutin-MNPs with Enhanced Magnetic Resonance Imaging and H2O2-Responsive Therapy of Alzheimer’s Disease in APPswe/PS1dE9 Transgenic Mice. Adv. Mater..

[27] Mishra S., Mishra S.R., Soni H. (2021). Efficacy of Hydrogel Containing Rutin in Wound Healing. EAS J. Pharm. Pharmacol..

[28] Zhao L., Qi X., Cai T., Fan Z., Wang H., Du X. (2021). Gelatin hydrogel/contact lens composites as rutin delivery systems for promoting corneal wound healing. Drug Deliv..

[29] Tran N.Q., Joung Y.K., Lih E., Park K.D. (2011). In Situ Forming and Rutin-Releasing Chitosan Hydrogels As Injectable Dressings for Dermal Wound Healing. Biomacromolecules.

[30] Farris A.L., Rindone A.N., Grayson W.L. (2016). Oxygen delivering biomaterials for tissue engineering. J. Mater. Chem. B.

[31] Camci-Unal G., Alemdar N., Annabi N., Khademhosseini A. (2013). Oxygen-releasing biomaterials for tissue engineering. Polym. Int..

[32] Alemdar N., Leijten J., Camci-Unal G., Hjortnaes J., Ribas J., Paul A., Mostafalu P., Gaharwar A.K., Qiu Y., Sonkusale S. (2016). Oxygen-Generating Photo-Cross-Linkable Hydrogels Support Cardiac Progenitor Cell Survival by Reducing Hypoxia-Induced Necrosis. ACS Biomater. Sci. Eng..

[33] Kang J.I., Park K.M., Park K.D. (2019). Oxygen-generating alginate hydrogels as a bioactive acellular matrix for facilitating wound healing. J. Ind. Eng. Chem..

[34] Patil P.S., Fountas-Davis N., Huang H., Michelle Evancho-Chapman M., Fulton J.A., Shriver L.P., Leipzig N.D. (2016). Fluorinated methacrylamide chitosan hydrogels enhance collagen synthesis in wound healing through increased oxygen availability. Acta Biomater..

[35] Li H., Wijekoon A., Leipzig N.D. (2013). Encapsulated Neural Stem Cell Neuronal Differentiation in Fluorinated Methacrylamide Chitosan Hydrogels. Ann. Biomed. Eng..

[36] Park S., Park K.M. (2018). Hyperbaric oxygen-generating hydrogels. Biomaterials.

[37] Hyun H., Yoo Y., Kim S., Ko H., Chun H., Yang D. (2019). Hydrogel-Mediated DOX⋅HCl/PTX Delivery System for Breast Cancer Therapy. Int. J. Mol. Sci..

[38] Darge H.F., Andrgie A.T., Hanurry E.Y., Birhan Y.S., Mekonnen T.W., Chou H.-Y., Hsu W.-H., Lai J.-Y., Lin S.-Y., Tsai H.-C. (2019). Localized controlled release of bevacizumab and doxorubicin by thermo-sensitive hydrogel for normalization of tumor vasculature and to enhance the efficacy of chemotherapy. Int. J. Pharm..

[39] Song H., Yang P., Huang P., Zhang C., Kong D., Wang W. (2019). Injectable polypeptide hydrogel-based co-delivery of vaccine and immune checkpoint inhibitors improves tumor immunotherapy. Theranostics.

[40] Newland B., Baeger M., Eigel D., Newland H., Werner C. (2017). Oxygen-Producing Gellan Gum Hydrogels for Dual Delivery of Either Oxygen or Peroxide with Doxorubicin. ACS Biomater. Sci. Eng..

[41] Motealleh A., Schäfer A.H., Fromm O., Kehr N.S. (2021). 3D-Printed Oxygen-Carrying Nanocomposite Hydrogels for Enhanced Cell Viability under Hypoxic and Normoxic Conditions. Biomacromolecules.

[42] Kumar R., Kehr N.S. (2022). Oxygen and Drug-carrying Periodic Mesoporous Organosilicas for Enhanced Cell Viability under Normoxic and Hypoxic Conditions. Int. J. Mol. Sci..

[43] Jensen C., Teng Y. (2020). Is It Time to Start Transitioning From 2D to 3D Cell Culture?. Front. Mol. Biosci..

[44] Zhu M., Wang Y., Ferracci G., Zheng J., Cho N.-J., Lee B.H. (2019). Gelatin methacryloyl and its hydrogels with an exceptional degree of controllability and batch-to-batch consistency. Sci. Rep..

[45] Riess J.G. (2009). Understanding the Fundamentals of Perfluorocarbons and Perfluorocarbon Emulsions Relevant toIn VivoOxygen Delivery. Artif. Cells Blood Substit. Biotechnol..

[46] Fang R., Jing H., Chai Z., Zhao G., Stoll S., Ren F., Liu F., Leng X. (2011). Study of the physicochemical properties of the BSA: Flavonoid nanoparticle. Eur. Food Res. Technol..

[47] Peng B., Li R., Yan W. (2009). Solubility of Rutin in Ethanol + Water at (273.15 to 323.15) K. J. Chem. Eng. Data.

[48] Advincula R.C., Dizon J.R.C., Caldona E.B., Viers R.A., Siacor F.D.C., Maalihan R.D., Espera A.H. (2021). On the progress of 3D-printed hydrogels for tissue engineering. MRS Commun..

[49] Sarker B., Singh R., Silva R., Roether J.A., Kaschta J., Detsch R., Schubert D.W., Cicha I., Boccaccini A.R. (2014). Evaluation of Fibroblasts Adhesion and Proliferation on Alginate-Gelatin Crosslinked Hydrogel. PLoS ONE.

[50] Liu H., Wu M., Jia Y., Niu L., Huang G., Xu F. (2020). Control of fibroblast shape in sequentially formed 3D hybrid hydrogels regulates cellular responses to microenvironmental cues. NPG Asia Mater..

