# Supplementary material for: 3D-Printable Oxygen- and Drug-Carrying Nanocomposite Hydrogels for Enhanced Cell Viability
Source: Nanomaterials (Basel). 2022 Apr 11;12(8):1304. doi: 10.3390/nano12081304 (PMC9028881; doi:10.3390/nano12081304)
Supplement: Supplementary file 1 [file nanomaterials-12-01304-s001.zip › nanomaterials-1662983-supplementary.pdf]

Supproting Information

3D-Printable Oxygen- and Drug-Carrying Nanocomposite Hydrogels for Enhanced Cell Viability

Ravi Kumar [1,2] and Nermin Seda Kehr [1,2] *

[1] Physikalisches Institute, Westfälische Wilhelms-Universität Münster, Wilhelm-Klemm-Straße 10, 48149 Münster, Germany; ravikapoorsaini@gmail.com
[2] Center for Soft Nanoscience (SON), Westfälische Wilhelms-Universität Münster, Busso-Peus-Straße 10, 48149 Münster, Germany

Loaded efficiency (E%) of rutin into PMO-PFC particles:

E% = (amount of rutin added-amount of free rutin) / (amount of rutin added) × 100 .
From calibration curve (Figure S1 A), Y= 0.01294x-0.00276
Absorbance of supernatant after loading of rutin into PMO-PFC (Figure S1 B), Y= 1.053667
Free rutin in supernatant in 1ml, x = 81.64 μg,
Amount of rutin added initially (80 mg) per 100 mg PMO-PFC, means 0.8 mg (800 μg) per 1 mg PMOPFC
Therefore E% = (800-81.64)/(800) * 100 = 89.79% ~ 90%.

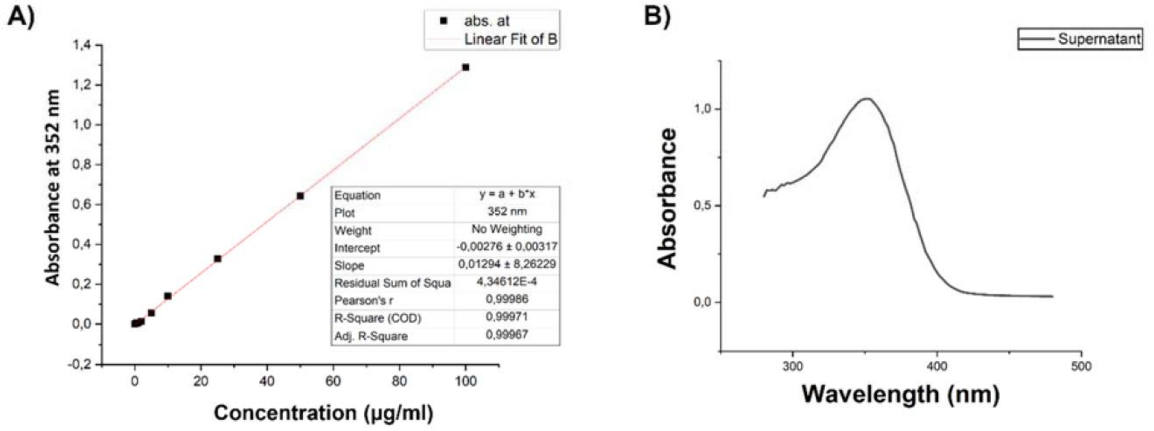

Figure S1. Calibration curve of rutin in water at wavelength of 352 nm (A). The absorbance spectra of supernatant after rutin coating of PMO-PFCs (B).

Similar way after PDL coating, the absorbance of supernatant was measured (Y = 0.7466) and rutin concentration was measured 58 μg/ml. therefore E % = (718-58 / 800)* 100 = 82.5% ~ 83%.

Calculation of rutin present in scaffold:

To calculate the initial concentration of rutin per scaffold before doing the release experiment, we calculate the rutin amount present in the volume filled by one hexagon template. For example, for Ru(PMO-PFC), the loading efficiency of rutin was 90% (i.e. 718 µg per 1 mg particle) and for Ru(PMOPFC)
PDL it was 83% (i.e. 651 µg per 1 mg particle), as already described in main text. The amount of particle was 1 mg/ml in the hydrogel mixture, therefore amount of rutin in each scaffold is the rutin present in the volume of the hexagon template (65 µl). For example, G+Ru(PMO-PFC) scaffold it is 46.7 µg (65*0.718) and for G+Ru(PMO-PFC)PDL scaffold it is 42.3 µg (65*0.651).

Swelling ratio of NC hydrogels:

NC hydrogels were placed in DI water for the given incubation time at 37 °C and weight (W) was measured before and after incubation.

$$\text{Swelling ratio} = \frac{(\text{W after} - \text{W before})}{W\ before}$$

Degradation of NC hydrogels:

NC hydrogels were placed in DI water at 37 °C and after incubation period the scaffolds were dried and weight (W) was measured before incubation and after drying.

$$Wloss\ \% = \frac{(\text{W before} - \text{W after})}{W\ before} \text{x } 100$$

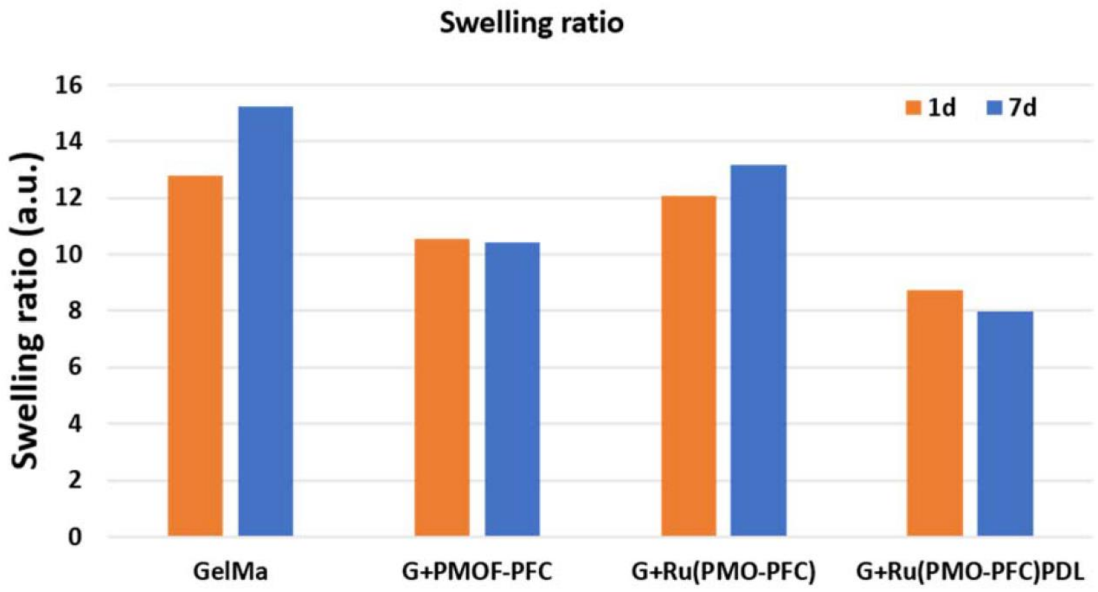

Figure S2. Swelling ratio of GelMa and NC hydrogels at 1day and 7 days.

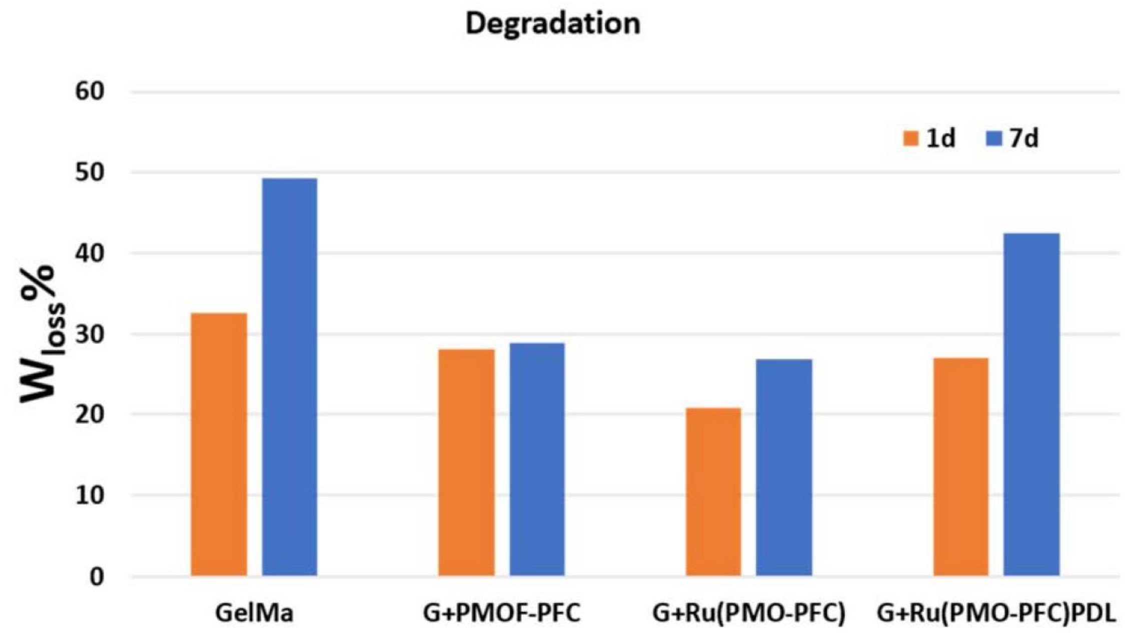

Figure S3. Degradation of GelMa and NC hydrogels at 1 day and 7 days.

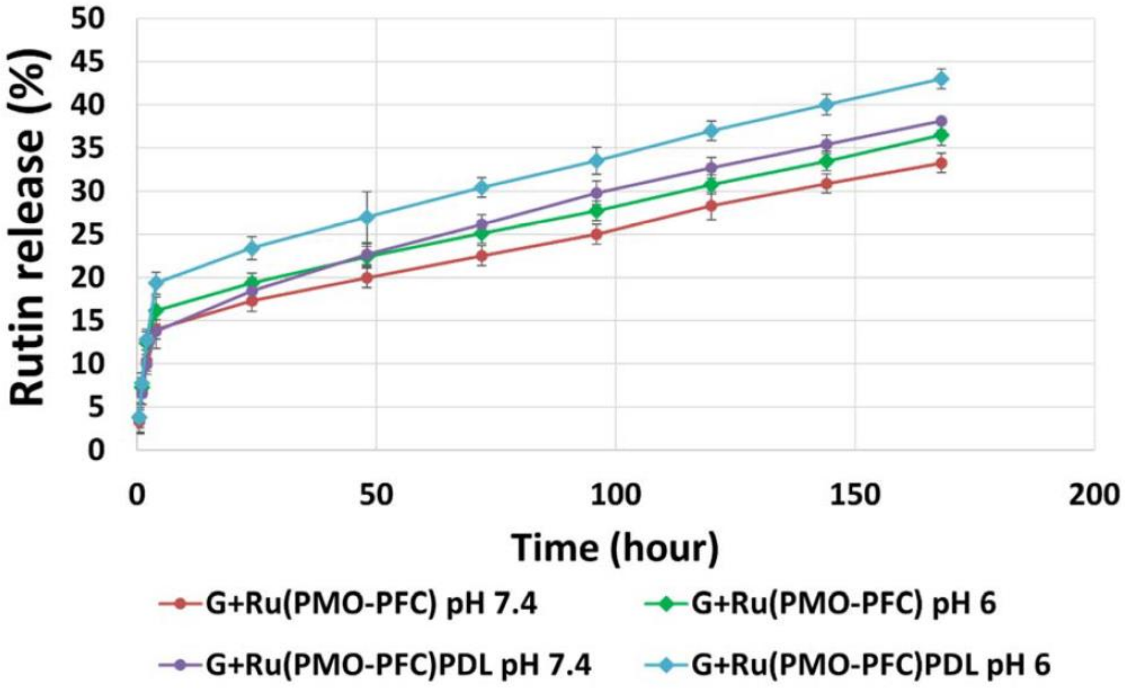

Figure S4. Rutin release in percentage for scaffolds at different pH value.
